# Poorly Understood Aspects of Striated Muscle Contraction

**DOI:** 10.1155/2015/245154

**Published:** 2015-04-16

**Authors:** Alf Månsson, Dilson Rassier, Georgios Tsiavaliaris

**Affiliations:** ^1^Department of Chemistry Biomedical Sciences, Linnaeus University, 39182 Kalmar, Sweden; ^2^Department of Kinesiology and Physical Education, McGill University, Montreal, QC, Canada H3A 2T5; ^3^Institute for Biophysical Chemistry, Medizinische Hochschule Hannover, 30625 Hannover, Germany

## Abstract

Muscle contraction results from cyclic interactions between the contractile proteins myosin and actin, driven by the turnover of adenosine triphosphate (ATP). Despite intense studies, several molecular events in the contraction process are poorly understood, including the relationship between force-generation and phosphate-release in the ATP-turnover. Different aspects of the force-generating transition are reflected in the changes in tension development by muscle cells, myofibrils and single molecules upon changes in temperature, altered phosphate concentration, or length perturbations. It has been notoriously difficult to explain all these events within a given theoretical framework and to unequivocally correlate observed events with the atomic structures of the myosin motor. Other incompletely understood issues include the role of the two heads of myosin II and structural changes in the actin filaments as well as the importance of the three-dimensional order. We here review these issues in relation to controversies regarding basic physiological properties of striated muscle. We also briefly consider actomyosin mutation effects in cardiac and skeletal muscle function and the possibility to treat these defects by drugs.

## 1. Introduction

**Figure 1:**
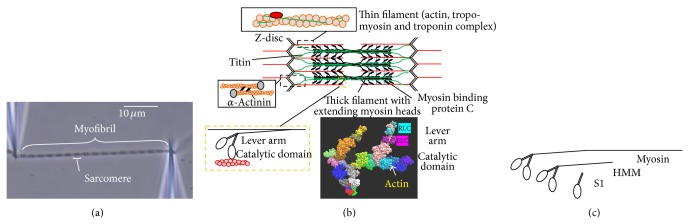
Hierarchical organization of myofibril. (a) Segment of myofibril captured between two microneedles for force measurements or application of length changes. (b) The sarcomere (structure between two Z-discs), with key protein components schematically illustrated. The resting length of the sarcomere is approximately 2.0 *μ*m in the human heart and 2.5 *μ*m in human skeletal muscle. Insets: the thin filament (top); critical molecular arrangement of the Z-disc (middle); extending myosin catalytic domain and lever arm interacting with actin filament (bottom; left) and 4 molecules of myosin subfragment 1 interacting with actin filament in the absence of ATP (PDB 1MQ8; bottom right). Regulatory light chain (RLC) and essential light chain (ELC) stabilize the lever arm. (c) Schematic illustration of myosin molecule (approximately to scale; see also (b)) and soluble motor fragments, heavy meromyosin (HMM) and subfragment 1 (S1) obtained by proteolytic cleavage under different conditions [356, 357].

Muscle contraction is the result of cyclic interactions between the contractile proteins myosin and actin, driven by the turnover of adenosine triphosphate (ATP) [1–8]. In vertebrate striated muscle (heart and skeletal muscle), actin and myosin are organized with several accessory proteins in highly ordered sets of interdigitating thin and thick filaments, respectively, forming repetitive 2.0–2.5 *μ*m long sarcomeres [2]. The functional units of muscle are the half-sarcomeres. These are connected in series to each other forming ~1 *μ*m wide myofibrils (Figures 1(a) and 1(b)) that run the entire length of the muscle cell (muscle fiber) and in parallel over the muscle fiber cross-section. During muscle contraction, globular myosin motor domains (heads) extend from the thick filaments to interact cyclically with actin binding sites on the thin filaments forming so-called cross-bridges (Figure 1(b)). The ordered arrangement on different hierarchical levels in muscles is highly beneficial to the effectiveness of the contractile process which is reflected in the independent evolution [9] of similar sarcomere organizations in phylogenetically distant organisms such as mammalians and Cnidaria (e.g., jellyfish). Some of the advantages of this arrangement are obvious, such as effective summation of length changes produced by sarcomeres arranged in series and forces over the muscle cross-section. However, there are likely additional, subtle benefits and even inbuilt imperfections of the ordered arrangement have been found to be of physiological importance [10–12].

Generally, there has been formidable progress [6, 13–16] in the understanding of striated muscle function since the elucidation of its basic principles [2, 17–19]. Initially, this progress relied mainly on mechanical and ultrastructural studies of muscle cells and biochemical studies of isolated actin and soluble myosin motor fragments. Key developments in the late eighties and early nineties transformed the field substantially with a shift of focus to a more reductionist approach (reviewed in [13]) and with complementary insights gained from studies of a range of newly discovered nonmuscle myosins. One of the major technical developments in this period was the in vitro motility assay [7, 20] where fluorescent actin filaments are observed [21] while being propelled by myosin or more often proteolytic myosin motor fragments (Figure 1(c)) such as subfragment 1 (S1) or heavy meromyosin (HMM). The latter contains two myosin heads, that is, two complete catalytic domains each with lever arm and two light chains, connected to a coiled-coil tail domain. Other key developments include (i) single molecule mechanics (optical tweezers based [22–24]) and single molecule fluorescence microscopy techniques [25] related to the in vitro motility assays, (ii) crystallization of actin [26] and the myosin motor domain (S1) [5, 27] allowing determination of their molecular structure with atomic resolution, and, finally, (iii) development of techniques for genetic engineering of myosin [28, 29]. While recent technical progress allowed a number of long-standing issues to be settled (cf. [30]), several important features of muscle contraction remain incompletely understood. This includes central issues such as (1) the molecular basis for the high maximum power-output [31, 32], (2) the mechanism of effective resistance to stretch of active muscle [33–35], and (3) the mechanisms by which myopathy mutations [36–43] and certain drugs affect muscle function.

The difficulties in addressing the problems (1)–(3) are due to limited understanding of important molecular mechanisms of the actomyosin interaction. This limitation is, in turn, attributed to challenges when integrating information derived from studies on different levels of hierarchical order (e.g., muscle cell versus single molecules) or using different techniques (e.g., biochemical solution studies versus muscle cell mechanics or single molecule mechanics). In this paper, we will review incompletely understood aspects of the actomyosin interaction. Other general aspects of muscle physiology and regulation are not included—the reader is instead referred to previous comprehensive reviews [15, 44, 45] and references therein.

## 2. The Molecular Basis of Muscle Contraction: Current View

Molecular motors may be classified as processive or nonprocessive depending on whether they take several steps or only one step along their track before detaching. A typical example of a processive motor is the nonmuscular myosin V with roles in certain forms of intracellular transport, for example, in the nervous system [46]. This motor is characterized by a slow and strongly strain-dependent detachment from the actin filament and appropriate coordination of its two motor domains (heads). Such characteristics allow the motor to move processively several steps along an actin filament.

The myosin II motor of muscle, generally denoted by “myosin” below, is, on the other hand, classified as nonprocessive. Thus, each myosin II motor domain spends most of its ATPase cycle time detached from actin and a single myosin motor is believed to take only one single step along an actin filament before detaching. Efficient operation of muscle therefore relies on a large assembly of myosin II motors working together. Consequently, the production of force and displacement by actomyosin in striated muscle is the result of cyclic interactions of billions of myosin motor domains with actin filaments. The efficiency and control of this process are optimized by the assembly of actin, myosin, and accessory (e.g., regulatory) proteins into highly ordered structures on different levels of hierarchical organization (Figure 1). The force-generating interaction cycles between actin and myosin are powered by the turnover of MgATP (denoted by ATP below) and are, except in response to rapid perturbations of length or tension [47–49], asynchronous between different motors as a basis for smooth shortening and force-development.

**Figure 2:**
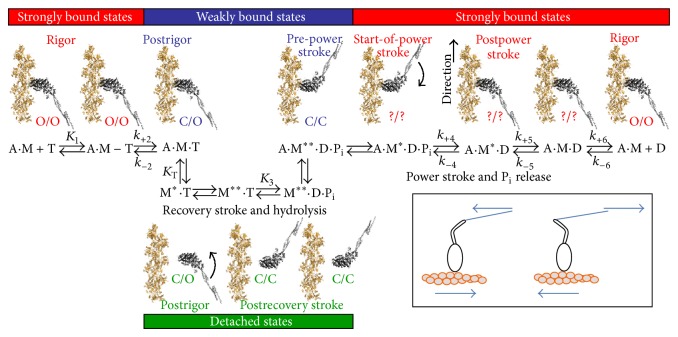
Simplified schematic representation of the predominant biochemical and structural states of the actomyosin ATPase cycle. Actin is depicted in orange; the myosin motor domain with artificial lever arm (X-ray structure PDB:1G8X [358]) is shown in grey colors (A = actin, M = myosin motor domain, T = ATP, D = ADP, and P = P_i_). The open (O) or closed (C) conformation of the active site elements switch 1 and switch 2 is indicated with switch 1 designated as the first. The power stroke corresponds to the switch 2 closed-to-open transition while the motor domain is bound to actin. The recovery stroke occurs in the detached state. It is assumed that the two heads of myosin act independently from each other and only one head is shown. Equilibrium constants and rate constants are denoted by upper case and lower case letters, respectively. Inset: Schematic illustration of tension in lever arm that causes muscle shortening (left) and that resists shortening (right).

The basic principles of the force-generating cross-bridge cycle in striated muscle have been elucidated on basis of biochemical, mechanical, and structural data [1, 2, 4–6, 23, 27, 47, 50–68]. Binding of ATP to the myosin motor domain first causes a structural change with a swing of the myosin lever arm (a “recovery stroke,” bottom Figure 2) that prepares the myosin head for executing a force-generating power stroke upon the next binding to actin. This event is also associated with altered disposition of loops (switch 1, switch 2, and P-loop) at the catalytic site (see further below). Subsequently, ATP is hydrolyzed to ADP and inorganic phosphate (P_i_) but the hydrolysis products remain bound to the active site of myosin. The subsequent, critical step in the force-generating cycle is the binding of the myosin head to the actin filament, forming a so-called cross-bridge. The initial binding is nonspecific [69, 70] and dynamically disordered with a range of azimuthal and axial angles of both motor domain and the light chain binding lever arm [71–73] relative to the actin filament. Furthermore, this initial weak binding is mainly electrostatic in nature [69, 70] with attached and detached states in rapid equilibrium. The transition from the initial weakly and non-stereo-specifically bound state of the myosin head to a stereospecifically bound state has been suggested to involve an average rolling transition of the myosin head on the actin filament [72–74] followed by locking in the stereospecifically attached state. This “roll and lock” transition may both increase the effective attachment rate [75] and contribute to the translation of the thin filaments [71] as well as the tension recovery following rapid length steps [72, 74].

Binding of the myosin head to an actin filament causes ~100-fold activation of the rate of P_i_ release. In the absence of actin, the P_i_ release, or rather a preceding conformational transition related to the reversal of the recovery stroke, is rate limiting for ATP turnover by myosin. The release of P_i_ from actin-bound myosin is associated with a strongly increased actomyosin affinity and a large drop in free energy. Further, there is an appreciable structural change that, in the absence or presence of a counteracting load, causes a swing of the light chain binding myosin lever arm or a tendency for such a swing, respectively. This swing of the lever arm, often termed the power stroke (Figure 2, step 4), is the basis for force-generation of muscle and the myosin induced sliding motion between the thin and thick filaments in the sarcomere. The type of structural change that actually occurs in this process depends on stretching elastic elements in the myosin head and elsewhere and, as just mentioned, the magnitude of the structural change varies depending on the external load on the cross-bridge (see further below).

Under certain physiological conditions, the muscle does not produce any mechanical power in spite of active cross-bridge cycles, such as during isometric contractions (without length changes), equivalent to isovolumetric contraction in a cardiac muscle contracting against closed valves. Further, eccentric contraction, when the muscle is stretched during activity, is associated with work done on the muscle rather than by the muscle [76]. Such eccentric contractions occur physiologically in skeletal and cardiac muscle [77]. During eccentric contractions, there is formation of actomyosin cross-bridges but the biochemical cycle in Figure 2 is not completed, as evident from the very low ATP turnover under these conditions [78]. Instead, the myosin cross-bridges are forcibly broken [34, 79–82] after pulling their elastic element backwards (corresponding to counterclockwise turning of lever arm in Figure 2). Thus, eventually, the backward pull leads to higher tension in the cross-bridge than sustainable by the actomyosin bond [81, 83–87]. The myosin head then detaches from actin, without release of ADP and subsequent rebinding of ATP, in contrast to the situation during shortening and isometric contraction. Indeed, cross-bridge detachment is also quite slow during isometric contraction, associated with key properties of the AM^∗^ADP state in Figure 2. This state has long been inferred in skeletal muscle [88–91] but its detailed properties were first studied more directly using different slow, nonstriated muscle myosins [92–99]. However, more recently a state with similar properties was found in skeletal muscle [61, 100]. It has also been essential to include in models of striated muscle contraction to accommodate findings that the ADP release occurs in two steps where the first step is an isomerization reaction that is accelerated by negative strain in the myosin lever arm (corresponding to clockwise turning in Figure 2). This results in opening of the nucleotide binding pocket (with a strain (*x*)-dependent rate constant *k*_+5_(*x*); cf. Figure 2) before ADP leaves rapidly with rate constant *k*_+6_.

The numerical value of *k*_+5_(*x*) is believed to be high for low *x*-values (dominating during rapid shortening), intermediate at intermediate *x*-values (dominating in isometric contraction), and very low for large *x*-values (dominating during forcible lengthening). Clearly, the AM^∗^ADP state and the strain-dependent transition *k*_+5_(*x*) are responsible for differences in cross-bridge kinetics between different conditions. For instance, at high physiological ATP concentration, if *k*_+6_ ≫ *k*_+2_ [101] and if *k*_+5_(*x*) is large (*k*_+5_(*x*) ≫ *k*_+2_), the overall dissociation constant *k*_*off*⁡_(*x*, [ATP]) is given by

(1) $$\begin{array}{c} k_{\mathrm{off}}\left(x,\left[\text{ATP}\right]\right)\approx \frac{k_{+2}\left[\text{ATP}\right]}{1/K_{1}+\left(k_{+2}/k_{+6}+1\right)\left[\text{ATP}\right]}. \end{array}$$

This applies for myosin heads that are brought into the drag-stroke region (*x* < 0 nm) during shortening where they resist sliding. In contrast, during isometric and eccentric contraction, when *k*_+5_(*x*) is small, then

(2) koff⁡x,ATP=k+2ATP1/K1+k+2/k+5xATP=k+5xATPk+5x/k+2K1+ATP.

In this connection it is of interest to consider the concept of duty ratio [102], *f*, that is, the fraction of the ATP turnover time that myosin molecules spend attached to actin. This ratio (see further [103, 104]) that is close to 1 for processive motors and <0.5 for nonprocessive motors is approximated by the following expression:

(3) $$\begin{array}{c} f=\frac{k_{\mathrm{on}}}{k_{\mathrm{on}}+k_{\mathrm{off}}}, \end{array}$$

where *k*_*on*⁡_ and *k*_*off*⁡_ are the cross-bridge attachment and detachment rate constant, respectively. Together with (1)-(2), this suggests that the duty ratio for myosin II while low during rapid shortening (e.g., ~0.05 in unloaded shortening) may be quite high in isometric and eccentric contraction as well as very slow shortening (with low *k*_*off*⁡_).

## 3. Poorly Understood Phenomena in Muscle Contraction

Because muscle contraction is due to the action of a very large ensemble of actomyosin motors it is necessary to use statistical models to relate contractile properties, such as force-velocity relationships, to actomyosin interaction kinetics and mechanics [1, 105–108]. Several models of this type have been developed, often incorporating the above-mentioned principles [31, 49, 109–111] and the additional assumption that all myosin heads act independently. The latter assumption deserves clarification. In most current cross-bridge models, it is assumed that (i) the myosin heads (even the two heads of one myosin molecule) do not compete for the same site on actin and (ii) the binding of one head does not affect the kinetics (nor its strain-dependence) for any transition involving another head (whether belonging to the same myosin molecule or not). This view is similar to the definition outlined in detail by Hill [105, 106]. It means that observable properties of muscle fibers can be readily obtained from mean values calculated from a given number of state probabilities without changing the model parameters between different conditions. For instance, if the myosin heads are assumed to act independently, it means that neither propagating structural changes in the thin filaments nor sequential actions of the two partner head are assumed to alter any parameter value such as attachment rate. Importantly, however, this independence does not prevent myosin heads from interacting (cooperating) by collectively altering the strain of other heads that are attached to a given surface or thick filament past which actin filaments move [1, 49, 112–114].

**Figure 3:**
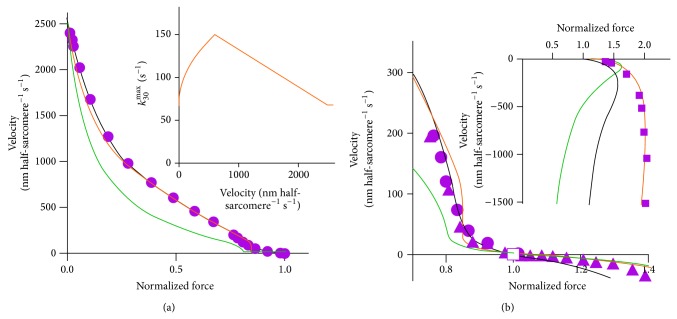
Force-velocity (load-velocity) relationship of frog muscle fiber. (a) Force-velocity relationship for shortening (positive velocity). Inset: assumed velocity dependence of attachment rate (*k*_30_^max⁡^) for certain model predictions in (a) and (b). (b) Force-velocity relationship for loads (forces) close to isometric (normalized force: 1.0) and for eccentric contractions (negative velocities). Inset: extended region for eccentric contraction. Purple symbols in (a) and (b): experimental data from [31] (circles), [191] (triangles), and [79] (squares). Green lines: model [110] with same attachment rate that would fit rise of tension in isometric contraction. Black lines: model [110] with attachment rate accounting well for the maximum power-output during shortening. Orange lines: model [110] with velocity dependent attachment rate for shortening (inset in (a)) and lengthening. In the latter case, the attachment rate constant increased from a maximum value of 67 s^−1^ in isometric contraction (inset (a)) to 335 s^−1^ at lengthening velocities ≥ 600 nm hs^−1^ s^−1^. Figures from Biophysical Journal [110] reprinted with permission from Elsevier/The Biophysical Society.

Statistical cross-bridge models with independent force-generators based on a kinetic scheme similar to Figure 2, but where each biochemical state exists for a range of elastic strains, account well for several contractile phenomena. This includes the information-rich relationship between velocity and load on a muscle (the load-velocity or force-velocity relationship; Figure 3; [115]). However, some key aspects of muscle function cannot be explained in a straightforward way by the most recent cross-bridge models. For instance, if the low myosin attachment rate to actin, suggested by the rate of rise of isometric force, is plugged into a statistical model (e.g., [1] or [31]), a velocity lower than that observed experimentally is predicted for shortening against intermediate loads. Thus, the high maximal power-output during muscle shortening [116] is not accounted for ([31, 32] and references therein; see also Figure 3). Neither is a low attachment rate consistent with the high steady-state resistance to forcible lengthening in eccentric contractions [79, 80, 110, 117]. Related to the difficulty in accounting for the high power-output during shortening, it is also difficult to account for the rapid repriming of the working stroke after a rapid length step, that is, the fact that the amplitude of the tension relaxation upon a rapid release recovers appreciably faster than tension after a priming release step. These phenomena may be reproduced by modifying existing models (e.g., [110]) to make the attachment rate velocity dependent so that it is low during isometric contraction, intermediate during shortening, and fast during lengthening [33–35, 79, 80, 110]. However, a velocity dependent attachment rate is not physically reasonable. Therefore, several alternative mechanisms have been proposed to account for the apparent velocity dependence of this rate [31, 35, 75, 110, 118–124]. Some of the alternative suggestions have kept the idea of independent myosin heads but appreciably increased the number of states and/or included the possibility for myosin heads to rapidly “jump” between neighboring sites on the actin filaments. Others have instead assumed important roles of cooperativity between the two heads of a given myosin molecule. However, there is still no consensus about these models. A third possibility is that the apparent velocity dependence of the attachment rate is related to myosin- or tension-induced structural changes along the actin filament [21, 125–133]. These could cooperatively alter the myosin binding properties of neighboring or even distant actin sites. The above considered alternative explanations are addressed in further detail below.

Another poorly understood issue is the pathogenesis of hereditary sarcomere myopathies which generally are due to single point mutations in myosin or regulatory proteins. The development of protein expression techniques for striated muscle myosin II [134–137] has enabled studies of the underlying functional deficit on the molecular level. However, the complex and multidimensional pathogenesis of the diseases [36–43] is the result of disturbed function on the whole muscle/heart level. This calls for new experimental approaches for studies of ensemble function on sarcomere or even super-sarcomere levels [43]. Whereas a reductionist approach will give important clues into the mechanisms of disease, a full understanding is likely to require studies on different levels of organization.

The challenges in understanding myopathies are very similar to those in understanding drug effects. Drugs with effects on muscle contraction are of interest for several reasons. For instance, there are compounds with activating potential [138, 139] that stimulate the actomyosin ATPase activity and enhance the contractility or even act as a kind of chemical chaperon that reactivates misfolded myosin molecules [140]. These substances represent a new generation of drugs, and improvements in their efficacy could provide new disease treatment strategies targeted against various hereditary myopathies, acute or chronical heart failure, and other forms of cardiovascular disease. Myosin inhibitors, on the other hand, could be useful for the treatment of myopathies caused by mutations in myosin that increase the active force while reducing the efficiency of muscle contractility [37, 141, 142].

More generally, any small chemical compound that effectively binds to the myosin motor domain and allosterically modulates the functional performance is of great interest in research on myosin and muscle [87, 100, 111, 143–157]. In contrast to point mutations, specific drug effects can be studied not only using expressed single molecules or disordered ensembles but also using muscle fiber preparations with maintained order of the myofilament lattice.

## 4. Different Experimental Systems

Below we consider why studies using different techniques and on different levels of hierarchical organization give results that are sometimes challenging to reconcile with each other (see also [158]). We also further consider model studies [1, 47, 52, 105, 107, 108, 112] because these have contributed to bridging the gap between different levels of hierarchical organization and integrated information from different experimental systems and from different scientific disciplines. Developments along these lines include studies [109, 117] attempting to integrate molecular structural models, biochemical schemes, and results from muscle mechanics. Furthermore, more recently, efforts have been made to achieve detailed fits of model predictions to a range of experimental data [31, 75, 103, 110, 111, 121, 122, 159].

Problems in integrating results of different types of studies are related to specific features and limitations that distinguish different experimental systems and approaches as outlined below.

### 4.1. Biochemical Solution Studies

Biochemical solution studies [4, 6, 50–54, 56–58, 63–65] have deciphered dominant parts of the kinetic scheme for the turnover of ATP by myosin and actin (Figure 2; reviewed in [16, 55, 57, 104]). Most of these studies have employed myosin subfragment 1 (S1) that contains the catalytic and actin binding sites of myosin and part of the lever arm (Figure 1(c)). Using this preparation, actomyosin states are generally probed under low ionic strength and unstrained conditions, corresponding to an elastic equilibrium position in muscle [107]. The lack of elastic strain is in contrast to the situation in muscle contraction where elastic strain is the basis for force-development and effects of force on actomyosin transition rate constants.

**Figure 4:**
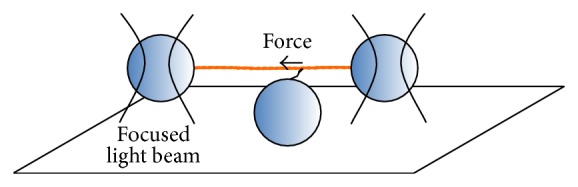
Optical trap system with actin filaments captured on two dielectric beads (optical traps) to which forces may be applied by a focused beam of near-infrared light. The actin filament held in the traps will interact with a single myosin motor on a third bead.

Whereas strain-dependent transitions cannot be probed in solution studies using S1, they can be studied in single molecule mechanics and in vitro motility assays where the myosin motor fragments are immobilized to surface substrates (Figure 4). Some aspects of strain-dependent transitions can also be investigated in solution using heavy meromyosin (HMM) motor fragments [99, 100, 160] because both of its motor domains can bind to actin filaments. This leads to strain between the heads although most likely different than that present in the ordered sarcomere lattice.

An interesting model, the so-called 3G model, was proposed in two influential papers [57, 58] based on evidence that myosin head binding to actin occurs in two steps. This led to the idea that each biochemical state (Figure 2) exists in three different structural states, with high, low, and very low affinity for actin. The 3G model furthermore assumes that the equilibrium between these states depends on the nucleotide occupancy of the active site. These ideas are relevant for the understanding of force-generation in muscle and have been incorporated into several more advanced statistical cross-bridge models (see below).

### 4.2. In Vitro Motility Assays and Mechanical Measurements from Small Ensembles and Single Molecules

In vitro motility assays may be viewed as extensions of biochemical solution studies with the key difference that the myosin motor fragments are immobilized to surfaces. Whereas the surface immobilization may affect protein function and complicate some aspects of data interpretation ([161, 162]) it ensures that strain-dependence of the actomyosin interaction is maintained. Therefore it also allows development of motion and forces. In vitro motility assay techniques [7] allow the observation of single actin filaments [21] when interacting with different numbers of myosin motor fragments and under different experimental conditions, for example, ATP concentration and ionic strength [163, 164]. This assay was later supplemented with a “laser trap” (“optical tweezers” technique [22–24, 68, 165, 166]). In this system, assumedly one myosin molecule attaches to an actin filament that is captured at the ends by beads “trapped” between two focused laser beams (Figure 4). Upon myosin-actin interactions, the displacement of actin filaments can be measured by tracking the position of the beads, showing that myosin II produces forces of 1–10 pN and maximum displacements of ~10 nm per interaction with actin [23, 94, 167–171]. In physiological conditions, however, the force and displacements produced by myosin and any other molecular motor are heavily influenced by the external load which dictates their functioning and mechanics.

The load dependence of the power stroke in single molecule studies has been investigated mostly in processive motors (e.g., myosin V) due to slow detachment kinetics and processivity, putting reduced demands on time resolution. The mechanics of myosin V has been studied when the motor was subjected to “pushing” and “pulling” forces, which corresponds to reduced and increased external load, respectively. The duration of attachments between the motor and actin filaments was decreased when the motors were pushed and increased when the motors were pulled [168, 172–174]. Furthermore, the attachment times were shortened with increasing ATP concentrations, suggesting that attachment was terminated when ATP binds to myosin following ADP release [168, 172–175]. Subsequent studies with myosins I and V and smooth muscle myosin II [94, 97, 98, 175, 176] suggest that increasing loads delay ADP release, resulting in a longer attachment time.

Single molecule mechanics studies using skeletal muscle myosin II [23, 169–171, 177, 178] are challenging due to high detachment rate and associated low* duty ratio*. Therefore, studying the effects of load of myosin II must occur during actomyosin attachments that are extremely short. A study performed with smooth muscle myosin, which has a longer average attachment time than striated muscle myosin, suggested that increasing loads increases this time [98]. Assuming that the total attachment/detachment cycle does not change during the actomyosin cycle, an increase in attachment time results in an increased duty ratio. The authors [98] also investigated the kinetics of the load dependence of attachment times and distinguished between two phases of attachment of myosin, consistent with structural studies showing two distinct myosin bond conformations: one conformation in the presence (phase 1) and the other in the absence (phase 2) of bound ADP. Increasing loads prolonged the duration of phase 1 but did not affect phase 2, suggesting that load dependence may be attributed to a transition between an actomyosin state with and without bound ADP (cf. *k*_+5_ in Figure 2). Later, using a laser trap system with improved time resolution [61], similar results were obtained using fast and slow myosin II from skeletal muscle.

The in vitro motility assays and related force-measuring techniques have answered a number of central questions with regard to striated muscle contraction suggesting that (i) only one myosin II head is necessary for production of motion and force [8, 179, 180], (ii) an unloaded displacement of 5–10 nm is produced by a myosin motor domain upon binding to an actin filament [23, 61, 67, 168, 171, 177] with the highest values in this range for two-headed myosin preparations and optimized orientations, (iii) a maximum force of about 10 pN is actively developed by a myosin motor domain [23, 170], (iv) there are target zones with sites, ~36 nm apart, along the actin filament to which an immobilized myosin II motor binds more readily [177, 181] than to other sites (see also [71, 74]), and (v) the displacement induced by a given strongly actin-attached motor domain occurs in two steps [61] where the second step is believed to be associated with the strain-dependent transition from the AM^∗^ADP to the AMADP state. Finally, recent developments [182] have allowed quite detailed probing of the force-dependence of several kinetic steps in the actomyosin turnover of ATP.

The importance of the surface-based assays is hard to overestimate. However, it is challenging to relate single molecule mechanics data to mechanics of muscle cells or myofibrils where very large ensembles of myosin motors interact simultaneously with the actin filaments (see below). Moreover, key aspects of muscle function such as the detailed shape of the force-velocity relationship and the apparent velocity dependence of the attachment rate constant have not been addressed because they result from interactions of a large ensemble of myosin motor domains with actin filaments in an ordered arrangement. In only few cases have the interaction between several (but not a large number of) motors and an actin filament been investigated using optical tweezers [183]. Furthermore, even if the interaction of a large number of myosin motors with actin filaments could be studied, it is difficult to assess cooperative phenomena properly. Such phenomena include that related to the role of the two myosin heads and their possible interaction with two actin filaments [160] or that due to an ordered arrangement of myosin motors in three dimensions around each actin filament.

Statistical and kinetic models of the type mentioned above (e.g., [1, 103, 108]) form an excellent basis for explaining results both from muscle cells, conventional in vitro motility assays and single molecule mechanical studies. However, there is risk of confusion when results from these different experimental systems are compared. This is exemplified by the myosin working stroke (power stroke) distance, as follows. First, we define this distance, *h*, as the average displacement of the actin filament actively produced when one myosin head binds to actin and completes its ATP turnover in the absence of counteracting load. The distance would be that measured in single molecule optical tweezers studies with low trap stiffness. It does not involve excursion of the myosin head elasticity into strains with negative forces (that resist sliding), that is, into the drag-stroke region [184]. This value for the working stroke would be identical to that: *h* = *v*_*f*_ *τ*_*on*⁡_(0) obtained from the in vitro sliding velocity (*v*_*f*_) and the myosin on time (*τ*_*on*⁡_(0)) at zero strain (e.g., measured in solution) if it is assumed that precisely one myosin head at a time propels an actin filament. These conditions imply immediate execution of a power stroke (to reach its zero-strain elastic equilibrium) upon myosin head attachment to actin and subsequent detachment with time constant *τ*_*on*⁡_(0) immediately followed, but not preceded, by attachment of a new myosin head and repetition of the cycle. Such ideal conditions are not fulfilled in reality. Therefore, the magnitude of the step length obtained from velocities measured in the vitro motility assay and optical tweezers studies differs by a factor up to ~2. This is clear by examining the condition with a very large ensemble of myosin heads that work together to propel the actin filament. This condition is fulfilled in muscle cells and approximately fulfilled in the in vitro motility assay if an actin filament is propelled over a surface with saturating density of myosin motor fragments. Under these conditions the elastic element of a large fraction of the myosin heads will be shortened to the extent that these cross-bridges resist sliding in the shortening direction (execute a drag-stroke). During steady-state unloaded shortening, the negative cross-bridge forces that counteract sliding are exactly balanced by the positive forces due to cross-bridges that undergo their power stroke. These force-levels are determined by the average strain of negatively (*v*_*f*_ *τ*_*on*⁡^−^_) and positively (〈*h*〉) strained cross-bridges, respectively, each factor multiplied by the cross-bridge stiffness. If the stiffness is Hookean the stiffness-values on the left and right sides of the equation cancel out and 〈*h*〉 = *v*_*f*_ *τ*_*on*⁡^−^_. This expression is deceivingly similar to that for *h*, given above. However, 〈*h*〉 is always smaller than *h* [103, 111, 184], generally 0.5*h* < 〈*h*〉 < *h*, consistent with *τ*_*on*⁡^−^_ < *τ*_*on*⁡_(0) which, in turn, is consistent with *τ*_*on*⁡_(*x*) = 1/*k*_*off*⁡_(*x*) (see (1)-(2)).

These relationships can be further expanded by considering also *h*_*∞*_ and *τ*_*on*⁡_ *∞*, defined as the average sliding distance and time, respectively, over which a given myosin head stays attached to the actin filament while executing first positive and then negative force (executing working-stroke followed by drag-stroke) in a large ensemble. Naturally, it also applies that *h*_*∞*_ = *v*_*f*_ *τ*_*on*⁡_ *∞*. Finally, it is readily shown (cf. [103]) that *τ*_*on*⁡_(0) > *τ*_*on*⁡_ *∞* > *τ*_*on*⁡_− and 2*h* > *h*_*∞*_ > *h* > 〈*h*〉 > 0.5*h*, where the last inequality is approximate.

### 4.3. Muscle Fiber Mechanics and Statistical Models

In the field of muscle mechanics, mechanical and optical sensor systems are used to relate length changes of muscle sarcomeres to the stiffness and forces developed by muscle cells (muscle fibers). The experiments can be performed either on intact [19, 185, 186] or on skinned [187–189] muscle fibers. The intact muscle cells are dissected from a living muscle using micromechanical tools (scissors, forceps, needles, etc.) leaving the cell membrane intact. In contrast, the membrane of skinned muscle cell segments is removed, chemically or mechanically, allowing the myofilament environment to be controlled from the bath fluid.

Of central importance in muscle mechanics are studies relating the imposed steady load on a muscle cell to the resulting steady velocity of the length change or equivalently the force developed upon imposition of a ramp shaped length change of a given velocity [1, 32, 115, 187, 190–195]. Force-velocity relationships obtained in either of these ways have demonstrated, although indirectly, that increased load increases the duration of the myosin power stroke [1, 196–198]. The velocity in response to increasing loads is continuously reduced, approximately according to a rectangular hyperbola [190] (however see [191]) from its maximum value in unloaded shortening to zero during isometric contraction (without length change). At this point the derivative of steady velocity versus steady load is continuous [191] when load increases above isometric force to cause lengthening with constant velocity (negative shortening velocity; Figure 3(b)). When a stretch is performed at low speeds (less than 2 muscle lengths s^−1^; *L*_0_  s^−1^), the increase in force during a length ramp has two components: (i) a fast phase, in which force increases substantially over a few nanometers per half-sarcomere and (ii) a slow phase, in which force increases a small amount or remains unchanged [79, 80, 86, 199–204]. The latter phase approximates the steady force during lengthening. The transition between these two phases occurs at a critical stretch amplitude of ~10 nm half-sarcomere, commonly associated with a critical strain of attached cross-bridges beyond which they are forcibly detached from actin [33, 34, 79, 80, 85, 86, 199–202, 204–206].

The mechanism behind the increase in force during stretch is still controversial. Several investigators have suggested that it is primarily due to an increased force per cross-bridge (increased strain) during stretch [33, 34, 80, 86, 207]. It has been made likely that this effect is caused by prepower stroke cross-bridges, in a state that precedes phosphate release [86, 201, 202, 208, 209]. Interestingly, in this connection, recent X-ray diffraction studies [124] suggested an increased fraction of non-stereo-specifically bound myosin heads during stretch, properties usually attributed to weakly bound prepower stroke cross-bridges. However, the idea of increased force-resistance being attributed to weakly bound myosin heads is not easy to reconcile with the above-mentioned critical strain of ~10 nm. Nevertheless, any model must account for the findings that the phosphate analogues vanadate and aluminium fluoride (AlF_4_), which are known to bias cross-bridges into a prepower stroke position, reduce isometric force of fibers treated with polyethylene glycol (which promotes myosin-actin interactions) considerably more than stretch forces [86, 208]. Similarly, the drugs butanedione monoxime (BDM) [207, 210] benzyl-toluene sulfonamide (BTS) [202] and blebbistatin [197], that are believed to inhibit main force-generating transitions, have similar effects. In this connection it is also of interest to mention that increased tonicity of the extracellular solution that causes volume shrinkage of intact muscle cells appreciably reduces the maximum isometric tension without affecting the maximum force during stretch [80, 204]. A similar result is seen at reduced temperature [211].

**Figure 5:**
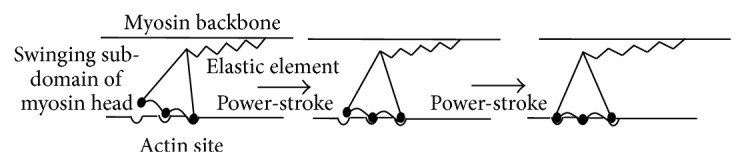
Model of the Huxley and Simmons type [47]. Force development is assumed to occur by thermally excited swing of a myosin head subdomain that stretches an independent elastic element. The swing is forward-biased (producing a power stroke) by progressively increased binding affinity between the myosin head and actin for each transition (to the right) that stretches the elastic element.

Many aspects of muscle mechanics have been strongly influenced by the pioneering work of AF Huxley from both a theoretical [1, 47] and experimental [19, 47, 212–215] perspective. Accordingly, muscle mechanical studies are often interpreted in terms of cross-bridge models that incorporate features of the Huxley and Simmons (1971; [47]) and the Huxley (1957; [1]) models. The latter model explains the basic steady-state properties of muscle (such as the force-velocity relationship) whereas the Huxley and Simmons (1971) model (Figure 5) accounts for the tension responses to rapid length changes imposed on a muscle cell. The combination of these two models accounts well for several aspects of muscle function [216].

The Huxley and Simmons model was inspired by the swinging cross-bridge model proposed by H. E. Huxley [2] on basis of ultrastructural evidence. Interestingly, in similarity to later results based on the atomic structure of myosin [5] the model incorporates ideas with an increasing number of attachment points between actin and myosin that stabilize high-force states. However, the model also raises critical questions. First, an independent elastic structure has not been unequivocally identified in the actomyosin cycle. Bending of the entire light chain stabilized alpha helical lever arm [217] or structural changes in the neighboring regions in the converter domain [141, 218] have been implicated to represent the elastic element (see also [219]). However, this region has also been implicated as the main component that swings during the force-recovery after a length step [66, 220].

This so-called swinging lever arm model followed the swinging cross-bridge model upon accumulating evidence against large-scale orientation changes of the entire myosin motor domain during force-generation [30] (however, see [71]). A second problem with the Huxley and Simmons [47] model is related to the number of states and force-generating structural transitions required. In their original paper, two stable attached states were assumed where transition from the low-force to the high-force state was accompanied by ~10 nm extension of the elastic element. As already was pointed out by the authors, two states are insufficient to account for the high power-output of muscle as well as for the rate of the tension transients using a model with an independent elastic element. This issue has become increasingly challenging after emerging evidence that the stiffness of the elastic element is somewhere in the range 1.7–3.3 pN/nm [32, 67, 221], considerably higher than previously believed.

Whereas a cross-bridge stiffness of ~1.7 pN/nm seems to be consistent with two tension generating steps [114, 222], a larger number of structurally and mechanically distinct states are required for a cross-bridge stiffness of ~3 pN/nm [75, 121, 122, 222, 223]. There is limited evidence for such a large number of states with different stable positions of the lever arm. Possibly, the issue would be resolved if the lever arm swing is preceded by a “roll and lock” transition of the entire myosin head that also contributes to force-recovery after a length step ([72]; see above). However, for any model with a large number of states, validation is difficult because a wide range of experimental findings can be reproduced with several free parameters whether the model is correct or not. A final complication related to the Huxley and Simmons [47] model is that the rates of relevant biochemical transitions observed in solution studies are considerably lower than the rates required to account for the rapid tension transients. This complication is related to the incompletely understood relationship between the rapid tension transients in response to length steps and the P_i_ release step in the ATP turnover by actomyosin, that is, the biochemical transition being most closely associated with the force-generating structural change in the actomyosin cross-bridge (see below). This is suggested by comparison of solution studies and rapid perturbations of contraction in intact and skinned muscle cells including rapid length steps (see above) and sinusoidal oscillations [89, 224, 225], rapid changes in load [48, 122], temperature (temperature jumps; [209, 225–231]), hydrostatic pressure (pressure jump; [232]), and phosphate concentration (phosphate jump; [151, 233–235]). Moreover, in skinned fibers, insight into the force-generating step and its relationship to, for example, P_i_ release (see below), has been obtained by investigating the [P_i_]-dependence of steady-state isometric tension and force-velocity data ([122, 192, 209, 236]).

An issue that has severely complicated the interpretation of a large number of muscle mechanical studies is the possibility of a nonlinear (non-Hookean) elasticity of the cross-bridges [67, 111, 237] and/or myofilaments [33, 238–242] or the presence of a time-invariant parallel-elastic element, possibly attributed to a fixed number of cross-bridges [243]. These issues (reviewed in [222]) have been considered further recently [244] but are not yet resolved making it challenging to interpret stiffness data in terms of the number of attached cross-bridges. This uncertainty is highlighted by experiments investigating the number of attached cross-bridges during shortening at different velocities [32, 85] and during slow stretch [33, 35, 120]. During shortening, stiffness measurements (after correction for presumed linear series elasticity) suggest that force and the number of attached cross-bridges are approximately proportional (at least at loads close to the isometric one) [32]. In contrast, an alternative approach for obtaining the number of attached cross-bridges based on the maximum tension response to very rapid stretches [206] suggests lack of such proportionality [85]. Furthermore, the assumption of linear filament elasticity suggests ([35] and later [120]) that the resistance to slow stretch of active muscle is mainly attributed to increased recruitment of cross-bridges. In contrast, Nocella et al. [33] found evidence for a nonlinear filament compliance suggesting the force-enhancement during stretch is mainly attributed to increased average cross-bridge strain (see also [34, 80]). Another type of studies that is not always easy to interpret is those based on time resolved low-angle X-ray scattering from contracting muscle cells. Whereas these studies have led to new important insights [217, 220, 238, 239, 245] there are conflicting views about the interpretation in some cases [246]. The interested reader is referred to other review-articles [247–249] for details.

Muscle fiber experiments have the advantage of maintained three-dimensional arrangement between the myofilaments in half-sarcomeres and preservation of accessory proteins that may affect contraction. On the other hand, the large number of protein components makes it necessary to use statistical models [1, 105] for interpretation of the experimental results and several different models are likely to account for a given data set. Furthermore, the interpretations of muscle mechanical and structural data (e.g., from low-angle X-ray diffraction) in terms of cross-bridge properties often rely on high degree of uniformity between half-sarcomeres along the length of a studied muscle fiber and over the muscle cross-section. In the absence of such order and uniformity, unpredictable emergent properties are possible. Model studies have suggested that the nonuniformities may cause residual force enhancement after stretch [250] and the suppression of oscillatory motion under certain conditions [43, 49]. Different types of nonuniformities between segments along muscle cells have also been observed experimentally [11, 19, 251–254] and found to play important physiological roles, for example, in speeding up relaxation after an isometric contraction [10] (see also [255, 256]) and contributing to aspects of the tension response to stretch [11, 12, 257].

The results of muscle fiber experiments may be affected, in unpredictable ways, by muscle fiber type, that is, by using fast or slow muscle (e.g., [61, 101, 258–260]) or due to mixtures of myosin isoforms in a given cell [251, 254] (see also [261, 262]). Furthermore, the level of activation and the presence of regulatory proteins ([151, 187, 263]) may affect the kinetics of the actomyosin interaction in different ways. Finally, a range of posttranslational modifications may affect function. This expanding field is not considered further here but it may be worth mentioning that the drug blebbistatin affected unloaded shortening velocity in skinned fibers differently in the presence and absence of phosphorylation of the regulatory myosin light chains [264].

### 4.4. Myofibril Mechanics

Myofibrils can be isolated both from skeletal and cardiac muscles and mounted for force-measurements and imposition of length perturbations (e.g., [207, 256, 265, 266]). Myofibrils are of particular interest to study because they are the smallest experimental units that maintain the three-dimensionally ordered myofilament lattice of striated muscle. The myofibrils are formed basically by sarcomeres arranged in series and with all major proteins intact (i.e., myosin, actin, troponin, tropomyosin, titin, and myosin binding protein C). Results from studies with myofibrils have been used to link studies on single molecules or proteins in solution with studies performed using muscle fibers. The length of myofibril segments to be studied can be virtually chosen by the investigators, and their diameter is substantially smaller (~1.0–1.5 *μ*m) than that of muscle fibers (~10 *μ*m). This is important, because it makes the diffusion time during activation of myofibrils very short, eliminating gradients of activation from the periphery to the core of the preparation. In contrast, the longer diffusion distances in muscle fibers can cause substantial gradients, not only in the activation level but also of ATP, ADP, and P_i_ concentrations, making interpretations at the actomyosin level complex.

The development of techniques for rapid solution exchanges during experiments with myofibrils enables exact determination of the rates of force development and relaxation during contraction, important indicators of the actomyosin interactions. Furthermore, the use of myofibril activation, in association with fast length changes imposed to the preparation, allows precise evaluation of the rate of force redevelopment (Ktr) following a shortening-stretching protocol [265] that was originally developed for application to muscle fibers [267]. The Ktr has been used effectively to define the kinetics of cross-bridges transiting between weakly bound and strongly bound states. The Ktr determined with high time resolution has been used not only for probing the steps of the actomyosin cycle, but also for comparison of myosin kinetics in muscles of different conditions, health, and disease (e.g., [268–270]). Finally, myofibril studies allow investigators to elucidate the detailed relation between force development, relaxation, and sarcomeres dynamics. Since myofibrils are formed by a single chain of sarcomeres, the force produced by the myofibril at both ends can only be produced and shared by these interconnected structures. Such structural geometry has been explored to infer the mechanical behavior of myofibrils upon activation and during/after loads that are imposed to the preparation [257, 266].

Recently, there have been studies using single sarcomeres [271] and isolated half-sarcomeres [272], preparations that by nature avoid sarcomere length nonuniformities, and thus open possibilities for investigations of contractile performance without confounding effects. The limitation of these preparations is their fragility—it is virtually impossible to activate single sarcomeres for more than 5-6 activation cycles.

### 4.5. Molecular Structure: X-Ray Crystallography and Cryo-Electron Microscopy

**Figure 6:**
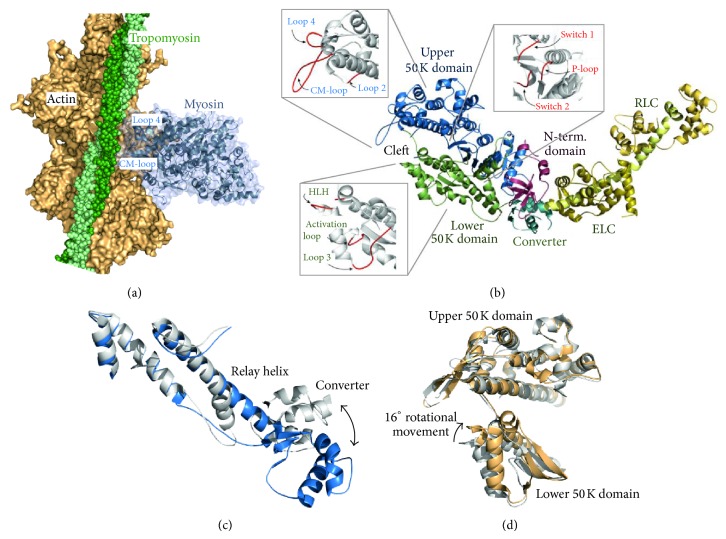
Structures of the rigor actomyosin complex and the myosin motor domain (S1) at different nucleotide states. (a) High resolution structure of the nucleotide-free actin-myosin- tropomyosin complex as obtained by cryo-electron microscopy (ref. [291], PDB IDs 4a7n, 4a7l, 4a7h, and 4a7f). (b) Ribbon representation of the atomic structure of chicken skeletal muscle myosin S1 fragment (PDB: 2MYS). S1 comprises 843 amino acid residues of the myosin heavy chain and two light chains (RLC and ELC) bound to the C-terminal neck region of the molecule. The central core comprises a seven-stranded *β*-sheet surrounded by several *α* helices. Characteristic is the deep cleft in the molecule. The cleft extends from the active site (P-loop, switch 1, and switch 2) to the actin binding elements, which are located in the upper (blue) and lower 50 K (green) domains. The N-terminus is adjacent to the C-terminus forming a protruding SH3-like *β*-barrel structure (red). The long C-terminal helix (light green) contains two IQ motifs that bind the light chains (ELC and RLC) and acts as a lever arm and conveys together with the converter domain local conformational changes to large movements. Highlighted in red in the insets are the actin binding and nucleotide coordinating loop and switch elements. (c) Conformational rearrangements of the relay helix (unwinding and kinking) and the converter (rotational movement) during the recovery stroke. The recovery stroke drives the detached myosin from the postrigor state to the prepower stroke state. The structures depicted are PDB ID: 2JHR and PDB ID: 1G8X. (d) Structural model for the strong binding start-of-power stroke (ref. [145]). The myosin power stroke is initiated by a transition from a weak-to-strong actin binding state. A rotational movement of the lower 50 K domain from the prepower stroke state (light grey, PDB ID: 2JJ9) enables a rigor-like strong binding geometry of the myosin at the actin interface (shown in brown ribbon representation) without changing the position of the converter domain. The structures were prepared with the PyMOL Molecular Graphics System, Version 1.7.2, Schrödinger, LCC.

Structural insights (Figure 6(a)) into the acto-myosin interaction have been obtained by combining crystallographic data for the myosin motor domain with information derived by electron microscopy and small angle X-ray scattering studies from myosin-decorated actin filaments [27, 59, 273–275]. The X-ray structures of the myosin motor domains crystallized so far fall into three categories dependent on the structural state they represent in the ATPase cycle (Figure 2). The distinctions are made on basis of the relative position of the active site elements (switch 1 and switch 2 closed or open; Figure 6(b)), the lever arm orientation (up or down), and the conformation of the actin-binding cleft (open, closed, or partially closed). The switch elements act as nucleotide sensors responsible for communication between the nucleotide biding pocket and the actin binding sites. Their reversible switching between two conformations opens and closes the active site around the *γ*-phosphate enabling hydrolysis and the coupling of internal conformational changes to larger rearrangements and rigid body movements of subdomains in the myosin motor that eventually lead to force generation. When considering states based on X-ray scattering and cryo-electron microscopy it is important to emphasize that they only capture metastable structural states.

The majority of the myosin structures crystallized with ADP.P_i_ analogs represent the prepower stroke state after ATP hydrolysis with weak affinity of the myosin cross-bridge for actin [157, 274, 276–279]. The cleft in most of these structures is partially closed. Further, switch 1 and switch 2 adopt closed conformations and the lever arm is in the up position. The second group of structures comprises states of the myosin motor domain assigned as postrigor [280–283]. These are thought to represent the prehydrolysis state (cf. Figure 2) of the myosin from which the recovery stroke takes place, transferring the motor to a catalytically competent prepower stroke state. In the postrigor states, the cleft is open, switch1 is closed, switch 2 is open, and the lever arm is down. A third group of structures, defined as rigor-like, have been obtained for myosin V and myosin VI [284–286]. According to the functional properties of these high duty ratio myosins, the crystallized states are thought to represent high actin affinity binding at a time after the power stroke has occurred. Characteristic for the majority of these nucleotide-free structures is a closed-cleft conformation and the lever arm down. Of relevance here, the rigor-like structure has also been obtained for muscle and nonmuscle myosin II [287, 288]. Despite the small differences seen in the extent and location of the cleft closure between the different rigor-like structures, it becomes apparent that cleft closure, although enthalpically unfavorable [289], is essential for facilitating the release of the hydrolysis products.

There is no crystal structure of the myosin motor domain bound to actin, but the rigor-like structures all exhibit features of an actin-bound state and high resolution cryo-electron microscopy support this view [290, 291]. Other limitations of available structural data are the lack of crystal structures showing states between the prepower stroke states and the rigor-like state.

In view of the limited availability of structural data, determining the sequence of events by which the myosin cross-bridge generates force has been made possible only by the combined analysis of structural information and biochemical data from solution kinetics together with model building including molecular dynamic simulations. In the absence of ATP, myosin forms a high affinity complex with actin (Figure 6(a)). In this strongly bound rigor state, the active site elements, switch 1 and switch 2, are thought to adopt an open conformation with the lever arm in a down position (Figure 2) [59]. The state subsequently turns into a low affinity state as Mg^2+^-ATP irreversibly binds to the myosin active site [292].

The Mg^2+^-ATP binding induces a closing of switch 1, which drives the formation of several new interactions such as a salt-bridge between switch 1 and switch 2 that assists in stabilizing the *β*-phosphate and enables the coordination of the Mg^2+^-ion and proper positioning of the surrounding water molecules for ATP hydrolysis. Kinetic studies with myosin mutants in which the formation of the salt bridge is disrupted are not capable of hydrolysis, emphasizing the critical role of the switch 1/switch 2 interaction [293–296]. At the same time, the active site rearrangements induced by Mg^2+^-ATP binding are coupled to the distortion of the seven-stranded *β*-sheet forcing the upper 50 kDa subdomain to undergo a large movement, which reduces the contact area and weakens the affinity to actin. This enables cleft opening and the full dissociation of the actomyosin complex [297]. The dissociated state is the hydrolysis competent state of myosin. According to current data, the hydrolysis reaction requires the closing of switch 2 [298, 299], which is coupled to larger rearrangements of the relay helix and the converter [300–302]. The 6 Å shift of switch 2—as seen between the postrigor and prepower stroke state structures (Figure 6(c))—causes a partial unwinding of the relay helix and a kink. Since the tip of the relay-helix is connected via hydrogen bonds and hydrophobic interactions with the converter, the relatively small switch 2 movement is amplified via the relay helix to a 65° rotation of the converter and a swinging of the lever arm from the initial down to the up position. This structural transition is known as the recovery-stroke [275, 282, 303–309].

The up position of the lever arm is the starting point of the force producing working stroke or power stroke, which requires rebinding of myosin to actin (Figure 2). Otherwise P_i_ is released from myosin without actin-binding, following a lever arm swing that represents the reversal of the power stroke and that is futile with regard to force-production [62].

Characterization of the actin binding elements by mutational analysis assumes that actin binding occurs sequentially through the contribution of at least six flexible myosin loops (Figure 6(b), close-up views) that modulate, in a nucleotide-dependent manner, the interaction strength and coupling to actin [296, 310–315] (see also [316]). According to solution kinetics, binding of myosin heads to the actin filament occurs in two distinct ways, weak and strong [57, 58, 317], that cannot entirely be explained by the current set of structures. The rigor-like structures allow predictions of how cleft closure induced by actin binding accelerates product release [284, 287]. However, what cannot be deduced from the rigor-like structural state are details of the conformational changes that initiate the power stroke and that accompany the transition from an initial weakly bound acto-myosin-ADP.P_i_ state to the actin-myosin rigor complex (Figure 2). A priori, there are several possibilities [62], including the presence of a start-of-power stroke state in which the myosin motor domain is strongly bound to actin and the lever arm is in an up position. Structure-based modeling of this putative state [318] suggests that the power stroke is not a reversal of the recovery-stroke, because the tight actin-binding constrains the relative motion of the upper and lower 50 K domain [59]. Rather, the power stroke is thought to be realized in at least two steps, involving a transition from the prepower stroke state to the proposed start-of-power stroke state. This could be accomplished by a rotational movement of the lower 50 K domain (Figure 6(d)), which subsequently closes the cleft thereby putting a torsional strain on the *β*4-strand of the central *β*-sheet via the W-helix forcing the molecule to subsequently straighten the relay helix, which in turn drives power stroke. The exact position of the active site switch elements in this transition state and their mutual interplay in the following states with respect to additional coupled rearrangements of the relay-helix and core *β*-strand cannot be accurately predicted from the current structures and models. With the help of kinetic studies, some speculations about the series of switch movements coupled to the power stroke can be made [319, 320]. However, additional structural and biochemical work is necessary to resolve the exact communication pathway that links actin binding to force production.

## 5. Poorly Understood Molecular Mechanisms in relation to Contractile Properties

An explanation for poorly understood phenomena in muscle contraction (Section 3) requires better understanding of the incompletely understood molecular mechanisms considered below.

### 5.1. Attachment of Myosin Head to Actin, Phosphate Release, and the Main Force-Generating Transition

There is currently rather incomplete understanding of the biochemical, mechanical, and structural events associated with myosin head attachment to actin and subsequent force-production.

Whereas we here focus on the force-generating transition it is of relevance to describe some uncertainties about the rate-limiting step for the ATP turnover cycle that has been placed somewhere between the ATP-hydrolysis and P_i_ release step (Figure 2) [16, 55, 65, 108, 321, 322]. The issue is important for explaining the increased apparent attachment rate during shortening against intermediate loads compared to isometric contraction (see above). For instance, if the attachment rate is limited by the rate of the hydrolysis process rather than by the attachment step or P_i_ release one may foresee higher apparent attachment rate during shortening. This is due to completion of the hydrolysis step during the time that actin target sites (with 36 nm separation) move past myosin heads that are incorrectly oriented for binding. This means that any sterically feasible cross-bridge attachment is faster under these conditions than during isometric contraction. The situation is similar if the rate-limiting step is between a so-called refractory and nonrefractory M.ADP.P_i_ state. This was the case in the model of Eisenberg et al. [108] and it is the basis for the capability of this model to account for the fast repriming of the power stroke by rapid reattachment of cross-bridges from a nonrefractory M.ADP.P_i_ state into a low-force state that is competent to undergo a force-generating transition upon a length step [118].

With regard to the relationship between the force-generating transition and P_i_ release, several issues are controversial. First, the major component of the fast tension recovery in response to length steps [47, 212, 225] is an order of magnitude faster than the tension responses to sudden changes in P_i_ concentration [233] hydrostatic pressure [232], temperature ([228–230] reviewed in [209]), and the force-generating process detected in spectroscopic studies [323]. Furthermore, the rate of tension recovery after length steps depended on the phosphate concentration after stretches but not after rapid releases. Thus, clearly the physical basis of the tension response to length steps and the other perturbations is not identical and the relationship between the length perturbation responses and phosphate release is complex. The idea of different molecular basis for the tension response to length jumps and temperature jumps is consistent with different structural changes according to X-ray diffraction patterns of skinned muscle fibers [217] but the relationship is complex. Thus, the tension response to temperature jumps seems to correspond to a slow phase of the tension relaxation in response to rapid length steps [225, 229, 230] and the overall rate of the tension response to length steps increases with temperature. The observed complexities (see also [230]) add to concerns [114, 158] that rather large number of states found necessary to account for the length-step response [122, 152] are not readily associated with states observed in biochemical and structural studies.

In order to elucidate the apparent incommensurability between results from different perturbation studies one may consider the characteristics of the observed tension responses in some detail. First, the dominant rate observed in the tension response to steps in pressure and temperature is rather similar [209] and the response to jumps in P_i_ concentration has a similar rate. Accordingly, temperature jumps have been claimed to affect an endothermic force-generating transition [209] in series with a rapid P_i_-binding equilibrium. Whereas most available data suggest that the force-generating transition occurs prior to the P_i_ release (see Table 1) there has been appreciable controversy about the exact temporal relationship (Table 1) and the possibility has also been considered that the P_i_ release is more or less loosely coupled to the force-generating transition [75, 121, 122]. Furthermore, whether force-generation occurs before or after phosphate release, there is controversy about the exact number of substeps and their rates [122, 151, 209, 229, 230, 236, 321, 323, 324]. If P_i_ release occurs before the force-generating transition (Table 1), then it seems that P_i_ release must be rate-limiting for force-generation because direct measurements of P_i_ release in solution [65] yield a similar rate as that attributed to tension generation following temperature jumps, phosphate jumps, and so forth. A slow P_i_ release has also been favored on basis of kinetic modeling [324], but in this case, the P_i_ release was believed to occur after the force-generating transition. Under such conditions, an AM.ADP.P_i_ state would be the main force-generating state and phosphate release would be rate-limiting for cross-bridge detachment. This is consistent with findings that an AM.ADP.P_i_ state is the dominant biochemical species in contracting myofibrils [325] and with spectroscopic studies of relay-helix motion in Dictyostelium myosin II catalytic domain [323]. The latter motion precedes a slower P_i_ release. However, numerous other findings suggest that P_i_ release is fast [122, 209, 236] and that an AM.ADP state (AM^∗^ADP in Figure 2) rather than and AM.ADP.P_i_ state dominates during steady-state contraction [89, 100, 326, 327].

Difficulties to reconcile results from experimental systems with different ionic strength, strain dependence, and so forth may contribute to the different views about the temporal relationship between P_i_ release and force-generation. The importance of strain, for instance, is reflected in 500-fold faster ^18^O exchange (reflecting P_i_-exchange) in isometric contraction of skinned fibers than in acto-S1 in solution [328]. Moreover, P_i_ release was inhibited during ramp stretches in cardiac muscle [329] and, finally, the P_i_-concentration affected the tension recovery after rapid stretches but not after rapid releases ([225]; see also [330]).

Another possibility is that the conflicting interpretations are due to models that do not capture certain critical features of cross-bridge operation. Furthermore, the lack of generality and stringency in definition of terms such as “main force-generating step” and “power stroke” contribute to the problems. These terms are used differently between researchers and between subfields such as muscle mechanics, single molecule mechanics, and actomyosin structural biology and biochemistry. The ambiguity is reflected in the discussion of the power stroke distance in Section 4.2 (see also [67, 114, 184, 222]).

**Figure 7:**
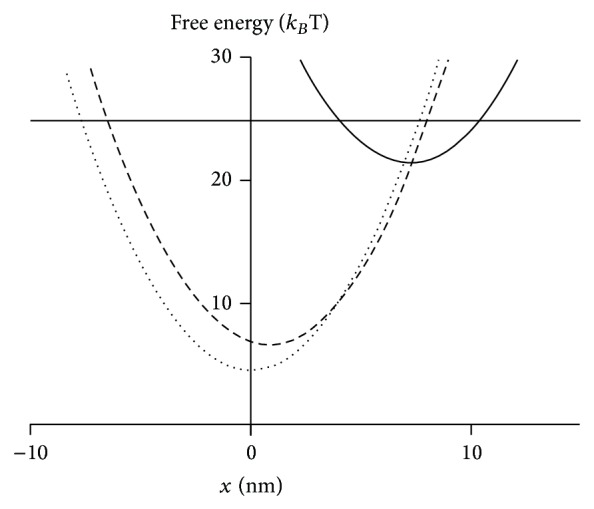
Free energy of main cross-bridge states in Figure 2 as function of the strain parameter *x*. Straight full line: detached states M.ATP and M.ADP.P_i_ lumped together into one state. Curved full line: AM.ADP.P_i_. Dashed line: AM^∗^ADP. Dotted line: AM, AM.ADP, and AM.ATP states lumped together. The parameter *x* = 0 when force is zero in the AM, AM.ADP, and AM.ATP states.

A direct identity of the force-generating transition associated with P_i_ release and length jumps was assumed in some early model studies [52, 107, 331] before the broad availability of data from perturbation studies other than length steps. The simplification was also used in recent models [111, 114] where the relationship between P_i_ release and force-generation was not in focus. In these cases, with key model states and their free energies illustrated in Figure 7, it is of interest to note that the model had high explanatory power accounting for both length-jump responses and a range of steady-state properties, for example, the force-velocity relationship, both in the presence and in absence of a drug affecting the strain-dependent transition from the AM^∗^ADP to the AM.ADP state [100, 110]. Later developments of the same model [111] also accounted for the effects of varying concentrations of ATP and ADP. Whereas temperature jump and P_i_-effects were not considered it was found that some temperature effects could be accounted for by increasing the free energy between the AM.ADP.P_i_ and AM^∗^ADP states [111]. These models could, however, not account for the high power-output of muscle during steady-state shortening or the high steady-state resistance to lengthening without assuming velocity dependent attachment rates (Section 3). Furthermore, due to the very fast detachment from pretension AM.ADP.P_i_ states, suggested by single molecule studies [182] and the associated weak actin affinity of these states, it seems unlikely that prepower stroke AM.ADP.P_i_ states can account for the high resistance to slow stretch. Thus, under slow stretches, the rupture force of a majority of the force-resistant cross-bridges seems to be high and with appreciable elastic strain [33, 81], seemingly incompatible with properties of AM.ADP.P_i_ states. However, future models must reconcile this finding with results (see above), based on effects of nucleotide analogs and drugs [197, 202, 208, 210], varied temperature, and altered P_i_-concentration [209], suggesting that prepower stroke cross-bridges in the AM.ADP.P_i_ state contribute appreciably to the stretch response.

**Figure 8:**
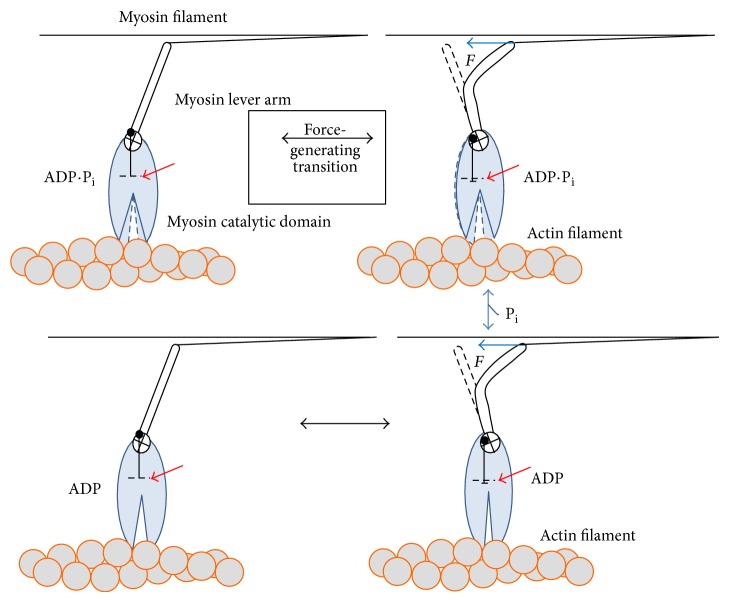
Tentative model. The elastic element is represented by bending of the lever arm being an integral part of the myosin head. This elastic element is stretched by a small amplitude structural change in the catalytic domain (from left to right). The schematic illustration is for isometric contraction. The position of the lever arm at elastic equilibrium of the main force-generating state (right) is illustrated by the lever arm drawn in dashed lines. The force-generating transition from left to right is orthogonal to the phosphate release step (vertical).

A further problem of current models for force-generation is, as pointed out above, that they assume an independent elastic element and require that the force-generating transition occurs in a large number of steps. An interesting alternative possibility, similar to that originally proposed in [107] is to assume an elastic element that is not independent from the swinging component and that is strained by a subnanometer structural change (in contrast to ~10 nm in the Huxley and Simmons model). This may be termed an Eyring like model [104, 110] where a local chemical change causes a transition into the new state, followed by ~10 nm relaxation of the elastic element into the minimum free energy of the new state. One model for how this could occur is schematically illustrated in Figure 8. Here, the localized structural changes strain the elastic element. The latter is here attributed to bending of the lever arm but the bending could also occur in the converter domain [141, 218]. The localized structural changes, on the other hand, involve thermal fluctuations of structural domains (e.g., related to relay helix, converter domain, central beta-sheet, and the loops around the nucleotide binding site), fluctuations that may precede P_i_ release [323]. A difference from the original Huxley and Simmons model [47] is the very small amplitude of the structural changes that lead to the high force-state in Figure 8, a fact that substantially reduces the energy barrier to be overcome. This Eyring mechanism [104] is in contrast to a more Kramers-like process, that is where a large scale diffusional straining of an elastic element against a load (as in the Huxley and Simmons [47] model) precedes the chemical change.

Something that further hampers our understanding of the force-generating transition is the fact that the atomic structural correlates of P_i_ release and force-generation in response to different perturbations are not well-defined. First, a question about the P_i_ release mechanism, including the exact time point in the ATPase cycle (does P_i_ release precede the power stroke or does the power stroke precede P_i_ release?), cannot be readily answered with the present structural models (see Section 4.5). On the other hand, structural information and computational analysis of the hydrolysis reaction postulate different equally feasible escape routes [62]. However, as touched upon above, a recent study based on solution kinetics and time-resolved fluorescence resonance energy transfer (FRET) experiments revealed that actin binding straightens the relay helix before phosphate dissociation assuming that the power stroke occurs before P_i_-release [323]. Structural details of the P_i_ release state of myosin are needed to understand how actin triggers product release and how active site switch elements rearrange to facilitate the P_i_ release.

The difficulty of crystallizing the actin-bound state of myosin has hampered detailed insights into the mechanism of force production. One possible way to overcome this problem would be the production of stable dimeric or trimeric actin oligomers. This minimal number of actin subunits could form a functional and crystallizable rigor complex for detailed analysis. In this way it would be possible to resolve, both the exact actin-myosin binding interface, the structural state and interactions between actin and the second myosin head [332], resolving the functional role of the latter. Optical trap experiments with native myosin II have shown that the degree of flexibility of the heads is sufficient to allow attachment to at least three subsequent binding sites on one actin filament [181].

In order to account for some of the apparently conflicting evidence it is interesting to consider ideas that each biochemical state exists in different mechanical/structural states in rapid equilibrium with each other [57, 58, 215, 333] (cf. Figures 5 and 8). More recently, these ideas have gained additional support [334] and been incorporated into rather complete cross-bridge models [75, 109, 117, 121, 122]. In the latter types of model each biochemical state in Figure 2 would be composed of several substates that differ mechanically and structurally by different extension of their elastic element, different degree of completion of the lever-arm swing and different affinity between actin and myosin (cf. Figures 5 and 8).

In terms of such a model, the tension response to length steps is due to very rapid transitions between mechanical/structural states (similar to those in Figure 5) without transitions between biochemical states (horizontal transitions in Figure 8).

The tension response to other perturbations, for example, temperature and phosphate jumps, is due to slower chemical reactions, for example, an isomerization prior to phosphate release that may or may not be strain-dependent.

Whereas models of this type account well for several experimental phenomena it will be important to limit the number of states to an absolute minimum and firmly define the properties of these states, including strain-dependence of interstate transitions, on basis of a range of different experiments. A further challenge will be to relate the structural course of events as defined by X-ray crystallography to the events seen in response to rapid perturbation experiments such as length-jumps, P_i_ jumps, and temperature jumps in muscle fibers. Possibly, mechanical experiments on different levels of hierarchical organization from single molecules over small ordered ensembles and myofibrils to muscle cells can help to bridge the gap if they are combined with spectroscopic techniques, for example, single-molecule FRET.

### 5.2. Number of Actin Sites within Reach for a Given Myosin Head

Several models of muscle contraction assume that only one binding site on the actin filament is within reach for binding of a given myosin head. This approximation allows simulation of most aspects of muscle contraction without severe limitations [110]. However, some additional phenomena may be accounted for if more binding sites are available. For steric reasons, such as the helical arrangement of the actin binding sites on the actin filament, it is likely that only groups of few (3–5) neighboring sites at 5.5 nm intersite distance are reached by myosin heads belonging to a given crown on the thick filament. This idea was supported by laser trap studies showing target zones for myosin binding corresponding to the helical repeat of 36–38 nm of the actin filament. The presence of neighboring sites on the same filament allows extension of the above models such as rapid reattachment to the neighboring actin binding site of a myosin head forcibly detached into an M^∗^ADP or MADPP_i_ state during shortening or stretch [75, 121, 122]. Such behavior may form the basis for an apparent velocity dependence of the cross-bridge attachment rate.

### 5.3. Role of Two Myosin Heads

The role of the second head in the dimeric myosin II molecule remains enigmatic, for example, whether the two heads are independent or cooperative in their interaction with actin and whether there is an alternating stepping behavior, where the heads subsequently bind to actin in a processive manner, thus enhancing force-output [160, 335–339].

Some studies have indicated that interhead cooperativity between the two heads of each myosin II molecule may not be important and the two heads are often viewed as independent force generators ([68, 171, 179]; see further above). However, there are a range of experimental results suggesting different forms of cooperativity of the two heads [74, 100, 124, 160, 168, 338, 340, 341]. One possibility is negative cooperativity between the two heads, that is, binding of one head to actin inhibits binding of the other [342] or one head prevents dissociation of the other head. Negative cooperativity of the latter type would enable maintenance of tension without energy consumption.

On the other hand, positive cooperativity has also been proposed [110, 160, 168, 341]. This may take different forms but one possibility is that binding of one head promotes binding of the other in a way that sequential actions of the two heads are promoted [110, 160]. Such effects may play a role in explaining the apparently faster attachment rate of cross-bridges during shortening and during lengthening. For instance, a mechanism with attachment of the second head was suggested [35], based on muscle X-ray diffraction data (see also [120]), to account for the effective resistance to lengthening. A similar model was proposed more recently [124], but with additional details, suggesting that a large fraction of the actomyosin cross-bridges during stretch are non-stereo-specifically bound (see also above). On the other hand, the idea that the appreciable resistance to stretch of active muscle is attributed mainly to increased number of attached myosin heads could not be corroborated in another recent study [33].

Modeling based on data showing two-headed attachment of fast myosin II [100] hinted [110] that sequential actions of the two heads of myosin II may become important in shortening against intermediate loads [60, 110] (intermediate velocities) when power-output is maximal. The rapid repriming of the working stroke [203] mentioned above has also been explained on this basis. Whereas recent in vitro motility assay tests [111] failed to corroborate the idea of sequential head actions they could neither falsify the hypothesis with certainty. Thus the predicted changes that were looked for were small and it is possible that the loss of cellular order in the in vitro motility assay was the reason for the failure to detect cooperativity [160]. Furthermore, effective interhead cooperativity may also require binding of the two myosin heads to neighboring actin filaments at interfilament distances similar to that in muscle [160] or there may be other forms of interhead cooperativity where the presence of two heads facilitates attachment of one of them but where sequential force-producing actions are not occurring [168, 340, 341].

### 5.4. Role of Structural Changes in Actin Filaments

The active role of myosin in force-production by actomyosin is universally accepted, but the actin filaments are generally [6], except for their allosteric activation of phosphate release from myosin, viewed as rope-like passive interaction partners. However, the actin filament structure is highly dynamic and altered during the force-generating process [21, 129, 132, 343, 344]. It is therefore reasonable to assume that structural changes in the actin filament are important for effective generation of force and power. Some authors have even proposed a dominant role of the actin filaments, for example, in providing gross structural changes that cause translation of actin over myosin or an asymmetric potential for biased diffusion of the myosin head. However, there is firm evidence for more facilitating and modulatory roles of the actin filaments. Thus, several studies [21, 127–133, 345, 346] suggest that myosin binding to actin or tension on the actin filament causes structural changes that propagate along the actin filament.

## 6. Understanding of Muscle Function Requires the Combination of Top-Down Disassembly and Bottom-Up Assembly of the Contractile Machinery

In addressing incompletely understood molecular mechanisms of muscle contraction, it will be critical to integrate results from experimental systems on different levels of hierarchical organization. It will also be essential to use a stringent joint terminology so that key concepts, such as “power stroke” have a similar meaning among muscle physiologists, biochemists, single molecule biophysicists, and structural biologists. Such an integrated approach should lead to models with a minimal number of states that integrate structural, biochemical, and mechanical information from fibers and single molecules, thereby laying a solid foundation for insight into poorly understood phenomena in normal muscle contraction as well as into effects of drugs and myopathy mutations.

Naturally, any cooperative interactions between myosin motors are lost in single molecule studies but detailed nonambiguous information about the strain-dependence of transition rates [61, 98, 182] is more readily derived using single molecules than ensembles. Such studies are now also possible using expressed myosin II from human striated muscle, both normal and with myopathy mutations [135]. On the other hand, the force-velocity relationship of striated muscle is a property of an ordered ensemble that cannot be fully characterized using single myosin motors. However, other challenges plague studies on muscle cells and myofibrils. Thus, there may be uncertainties about interpretation of mechanical and structural data from muscle fiber and myofibril mechanics due to incompletely characterized elastic components, emerging properties in the large ensemble of motors [347] that are not readily extrapolated back to actomyosin interactions or related effects of sarcomere nonuniformities. Clearly, it would be of great interest with new types of experiments to bridge the gap between the ordered system and single molecule studies. One may consider two complementary approaches. First, top-down disassembly, or rather a combined top-down disassembly and bottom-up assembly, of the contractile systems may be achieved by starting with skinned fibers or myofibrils [66, 348, 349] and removing more and more components, possibly followed by reconstitution. The other approach is pure bottom-up assembly of ordered contractile systems from single molecules. Simple versions of such experimental systems, for example, using different number of myosin motors randomly adsorbed to surfaces [183, 350] or incorporated into synthetic myosin filaments on a pedestal [67, 171], would allow studies of force-velocity relationships of ordered and disordered ensembles of myosin motors with one or two heads [179] interacting with actin filaments. Next, it may be of relevance to add other protein components one molecule after the other such as troponin and tropomyosin and protein C.

On more advanced level one may consider the use of DNA-origami scaffolds (cf. [351]) and engineered attachment points of the motors [135] to produce well-defined ordered arrays. Furthermore, if the scaffold could be produced inside a hollow nanowire [352] it may even be possible to build up in vitro assay systems with maintained 3D order similar to that in the myofilament lattice. Finally, it is of interest to use extensively engineered myosins [353–355] to investigate specific properties.

Interesting questions that may be possible to answer by combining single molecule studies and modelling with new experimental systems featuring few to many protein components in different ordered arrangements would be the following.

- Overall, are there emerging properties when going from single molecules to ordered ensembles or are the ensemble properties fully characterized on basis of single molecule components?
- What is the role of the two heads in single molecules and in ordered ensembles of different size?
- What is the role of 3D order, for example, the possibility of a given two-headed myosin motor to interact with two or more actin filaments at similar inter-filament distances as in a muscle cell?
- What are the roles of different accessory proteins?
- What is the importance of variations between half-sarcomeres in overlap between the thick and thin filaments and other variability in contractile properties over the fiber cross-section and along the length of a myofibril?
- What are the mechanisms determining the force-velocity relationship, for example, the role of two-myosin heads, structural changes in actin filaments, 3D order, and single myosin heads rapidly “jumping” from one actin site to the next when they are part of an ensemble. These issues may be accessible by studying force-velocity relationships of ordered ensembles of different sizes, using one-headed and two-headed myosin fragments and myosin motors with critical mutations, for example, affecting attachment properties or P_i_ release.
- Can ensemble effects of myopathy mutations and drugs be predicted from single molecule studies and solution biochemistry?

## Figures and Tables

**Table 1:** Conflicting evidence from experimental studies and theoretical considerations related to temporal relationship between P_i_ release and force-generating step in actomyosin cross-bridge cycle in muscle.

Force-gen. before P_i_ release	P_i_ release before force-gen.	Loose coupling
Kawai and Halvorson [89] P_i_ release fast	Davis and Epstein [230]. L-jumps and T-jumps in skinned muscle fibers	Caremani et al. [122]. Load-clamp expts. in skinned muscle fibers at varied [P_i_]

Dantzig et al. [233] based on P_i_ jump experiments in skinned muscle fibers P_i_ release fast	Davis and Rodgers [229]	

Tesi et al. [235] P_i_-jumps and tension versus [P_i_] in myofibrils P_i_ release fast	Spudich [16]	

Smith and Sleep [324] based on comparison of kinetic models P_i_ release slow	Sweeney and Houdusse [60] from reviewing structural data from several studies	

Ranatunga [209] from reviewing own work and work by others P_i_ release fast	Conibear et al. [359]	

Caremani et al. [236]. Contraction of skinned fibres at different [P_i_] P_i_ release fast		

Muretta et al. [323]. Spectroscopy applied to Dictyostelium myosin II P_i_ release slow		
